# Where Comity in Science Goes to Die

**DOI:** 10.1016/j.jacbts.2024.11.003

**Published:** 2024-12-23

**Authors:** Douglas L. Mann

**Figure:**
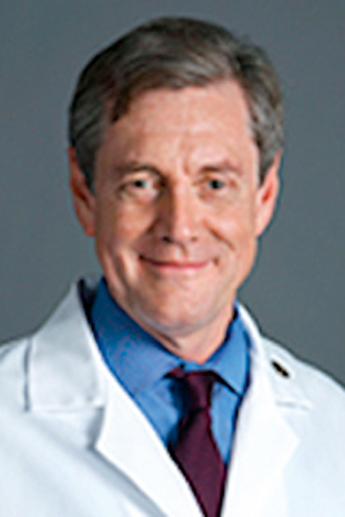

Comity, the courtesy and respect that allows for constructive discourse, has long been a cornerstone of civil society. In both public and scientific arenas, civil discourse fosters dialogue in which ideological and intellectual differences can coexist symbiotically, if not productively. However, in recent years, this shared respect has eroded, turning interactions into confrontations, rather than meaningful discussions, thereby challenging our ability to pursue progress. Even a cursory glance at the current state of American politics reveals just how much civility has declined. Political discourse, once an open forum in which leaders debated policies with a shared commitment to public welfare, has become a battleground of personal grievances, ad hominem attacks, and distortions of the truth.

The decline in comity may have also reached a tipping point in the scientific community, creating an atmosphere in which scientists often find themselves constrained to ideological bubbles. Heightened polarization and a pervasive attack culture discourage researchers from openly discussing findings that could provoke a backlash, leading them to engage only with like-minded colleagues. This isolation stifles intellectual diversity and inhibits the open exchange of ideas vital for scientific advancement.

Several factors contribute to the decline of scientific comity, including hypercompetitive funding environments or the fear of being scooped by rival labs. To this list, I suggest the possibility that if life imitates art, then science, in some respects, imitates societal norms. Disagreements about a scientific finding are often perceived as threats, rather than an opportunity to better understand the implications of a new finding, further eroding the spirit of open discourse that is essential to scientific progress.

The politicization of science, especially in areas like vaccine and public health research, has further strained collegiality. As this rhetoric has taken hold, scientists have faced increasing hostility, as exemplified by the threats and harassment experienced by Dr Anthony Fauci. Fauci, who led the public health response to the COVID-19 pandemic in the United States, received multiple death threats that required a security detail to protect both him and his family. When physician-scientist civil servants who have dedicated their entire lives to public health are turned into targets, rather than regarded as respected advisors, the wheels of scientific progress are inevitably doomed to come off the wagon.

The broader implications of this decline in comity are significant. Science, inherently collaborative, depends on diverse ideas intersecting to drive discovery. When scientists retreat into their own conceptual echo chambers where ideas are reinforced and rarely challenged, scientific innovation suffers, insofar as it erodes the collaborative spirit on which science thrives.

## Restoring Comity: A Path Forward

While the decline in comity presents significant challenges, there are steps that individuals and institutions can take to foster a culture of respect and civility. In the political sphere, leaders must prioritize integrity over popularity, committing to engage in substantive, fact-based discourse, rather than succumbing to sensationalism. Media outlets, too, have a role to play; by resisting the temptation to cater exclusively to partisan audiences, they can help bridge informational divides and encourage viewers to consider multiple perspectives.

In academia, fostering a culture of collaboration and intellectual humility is essential. Scientific progress relies on the ability to question, revise, and sometimes abandon long-held beliefs when new evidence emerges. This is hard to achieve when investigators only feel safe in their own conceptual headspace. Universities and research institutions should emphasize the importance of open dialogue and constructive criticism that create an environment in which researchers feel comfortable engaging in honest debate, even when their viewpoints may not be mainstream.

## Conclusions

The decline of civility in scientific discourse is a profound challenge that is driven by complex forces, including societal polarization, media influences, and the increasing pressures that investigators face. Comity in science dies within ideological bubbles that form when investigators no longer feel comfortable/safe discussing opposing points of view. This is particularly true for translational research that requires teams of scientists from different backgrounds to work together. Scientific journals can play a vital role in breaking down these barriers by actively encouraging diverse perspectives and publishing constructive editorial comments that accompany papers that have provocative findings that challenge prevailing scientific dogmas. By fostering open debate and rigorous peer review in a respectful manner, journals can create a space in which researchers feel safe to engage across ideological divides, helping to restore a spirit of comity in science. At *JACC: Basic to Translational Science*, the editors strive to maintain an open dialogue with authors, readers, industry, regulators, and the public in order to foster progress rather than division. To this end, we welcome your thoughts on how we can enhance civility in scientific discourse, either through social media (*#JACC:BTS*) or by email (jaccbts@acc.org).

